# User Outcomes for an App-Delivered Hypnosis Intervention for Menopausal Hot Flashes: Retrospective Analysis

**DOI:** 10.2196/63948

**Published:** 2025-01-09

**Authors:** Katherine Scheffrahn, Claire Hall, Vanessa Muñiz, Gary Elkins

**Affiliations:** 1 Mind-Body Medicine Lab Department of Psychology and Neuroscience Baylor University Waco, TX United States; 2 Mindset Health Melbourne Australia

**Keywords:** hypnosis, hypnotherapy, hot flash, menopause, women’s health, gynecology, smartphone app, applications, mobile health, mHealth, user, outcome, intervention, alternative, complementary, mind-body, mobile phone

## Abstract

**Background:**

Hypnotherapy has been shown to be a safe, nonhormonal intervention effective for treating menopausal hot flashes. However, women experiencing hot flashes may face accessibility barriers to in-person hypnotherapy. To solve this issue, a smartphone app has been created to deliver hypnotherapy. The Evia app delivers audio-recorded hypnotherapy and has the potential to help individuals experiencing hot flashes.

**Objective:**

This study aims to determine user outcomes in hot flash frequency and severity for users of the Evia app.

**Methods:**

This study is a retrospective analysis of a dataset of Evia app users. Participants were divided into 2 groups for analysis. The first group reported daytime hot flashes and night sweats, while the second group was asked to report only daytime hot flashes. The participants in the first group (daytime hot flashes and night sweats) were 139 women with ≥3 daily hot flashes who downloaded the Evia app between November 6, 2021, and June 9, 2022, with a baseline mean of 8.330 (SD 3.977) daily hot flashes. The participants in the second group (daytime hot flashes) were 271 women with ≥3 daily hot flashes who downloaded the Evia app between June 10, 2022, and February 5, 2024, with a baseline mean of 6.040 (SD 3.282) daily hot flashes. The Evia program included a 5-week program for all participants with daily tasks such as educational readings, hypnotic inductions, and daily hot-flash tracking. The app uses audio-recorded hypnosis and mental imagery for coolness, such as imagery for a cool breeze, snow, or calmness.

**Results:**

A clinically significant reduction, defined as a 50% reduction, in daily hot flashes was experienced by 76.3% (106/139) of the women with hot flashes and night sweats and 56.8% (154/271) of the women with daily hot flashes from baseline to their last logged Evia app survey. On average, the women with hot flashes and night sweats experienced a reduction of 61.4% (SD 33.185%) in their hot flashes experienced at day and night while using the Evia app, and the women with daily hot flashes experienced a reduction of 45.2% (SD 42.567%) in their daytime hot flashes. In both groups, there was a large, statistically significant difference in the average number of daily hot flashes from baseline to end point (women with hot flashes and night sweats: Cohen *d*=1.28; *t*_138_=15.055; *P*<.001; women with daily hot flashes: Cohen *d*=0.82; *t*_270_=13.555; *P*<.001).

**Conclusions:**

Hypnotherapy is an efficacious intervention for hot flashes, with the potential to improve women’s lives by reducing hot flashes without hormonal or pharmacological intervention. This study takes the first step in evaluating the efficacy of an app-delivered hypnosis intervention for menopausal hot flashes, demonstrating the Evia app provides a promising app delivery of hypnotherapy with potential to increase accessibility to hypnotherapy.

## Introduction

### Background

During the menopause transition, approximately 80% of women experience hot flashes, and approximately 20% of women report hot flashes to be very bothersome [1,2]. Hot flashes negatively impact women’s quality of life [3], and because the period during which women experience hot flashes lasts an average of 7.4 years, the impacts are long lasting [4].

In the past, the standard treatment for hot flashes, both premenopausal and postmenopausal, has been hormone therapy. However, hormone therapy has been associated with an increased risk of cancer, strokes, and thromboembolism [5-7]. Due to these concerns, many women do not wish to use hormone therapy; moreover, experts do not recommend hormone therapy due to these potential health issues [8].

Most of the options for nonhormonal treatments are either not as efficacious as would be desired or increase the burden on women significantly. Selective serotonin reuptake inhibitor, serotonin-norepinephrine reuptake inhibitor antidepressants, gabapentin, and paroxetine mesylate are nonhormonal medications approved by the US Food and Drug Administration for reducing hot flashes, but all these medications are associated with negative side effects [9-15]. A medication newly approved by the US Food and Drug Administration for menopausal hot flashes, fezolinetant (Veozah), has demonstrated evidence of effectiveness in reducing hot flashes [16,17]. However, fezolinetant’s potential side effects include liver injury; to guard against this, patients must undergo routine blood work during the first 9 months of using this drug [17]. This risk of liver injury and the blood work required places undue burden on women seeking a beneficial nonhormonal treatment for hot flashes.

When turning to nonpharmacological, nonhormonal intervention options, a commonly used intervention is cognitive behavioral therapy, which does decrease the level of distress women feel from hot flashes; however, cognitive behavioral therapy does not reduce the number of hot flashes experienced [18,19].

Clinical hypnotherapy is a mind-body intervention that has been shown to be effective in reducing both the frequency and severity of menopausal hot flashes [15,20,21]. Hypnosis is a state of consciousness involving focused attention and reduced peripheral awareness characterized by an enhanced capacity for response to suggestion [22]. Hypnosis is a safe, nonhormonal, nonpharmacological treatment with minimal to no adverse events [15,20,21].

However, face-to-face hypnotherapy for hot flashes has limited accessibility for many women. Challenges such as financial concerns, a lack of proximity to a hypnotherapist, or the time commitment needed for appointments may all factor into women being unable to access hypnotherapy [23,24]. A smartphone app-delivered hypnotherapy intervention for menopausal hot flashes can help overcome these intervention implementation barriers. Mobile delivery of hypnotherapy provides convenient and affordable hypnotherapy for women experiencing hot flashes. With the significant growth of the mobile health industry, smartphone apps have become a commonly used method for intervention dissemination, and this approach can also be used to expand access to hypnotherapy for those in need [25].

To this end, a smartphone app to deliver hypnotherapy for hot flashes has been developed. Created by Mindset Health, the Evia app delivers hypnotic inductions and educational information for hot flashes. The Evia program was developed based on the hypnosis protocol for hot flashes tested in multiple randomized clinical trials [20,21]. The app offers a 5-week program with daily tasks such as educational readings, hypnotic inductions, and hot flash tracking. The app uses audio-recorded hypnotherapy and mental imagery for coolness (ie, imagery for cool breeze, snow, and calmness).

The Evia app holds significant potential to help individuals experiencing hot flashes and has already greatly increased women’s access to hypnotherapy for hot flashes due to its availability through smartphone app stores. However, research on the app’s effectiveness is lacking. A previous study analyzed Evia app users’ characteristics and demographics and demonstrated that the age of Evia app users on average is consistent with the age of menopause onset and the emergence of menopausal symptoms [26]. In addition, this previous study demonstrated that on average app users experienced ≥5 hot flashes per day, a frequency similar to that of participants in previous randomized clinical trials.

### Objectives

This study, which will be the first to provide data on Evia app users’ outcomes, is a retrospective analysis of data collected from participants who downloaded and used the Evia app. The data, collected by Mindset Health, are from the questionnaire that users fill out before beginning the program, as well as the hot flash logs that users are encouraged to fill out throughout the program. There were 2 groups of participants. The first group was asked about daily hot flashes experienced during the day and night sweats (hot flashes that occur at night), while the second group was asked to report only daily hot flashes (daytime hot flashes). The data from each group were separately analyzed to fulfill this study’s aims.

This study aims to (1) determine self-reported user outcomes regarding whether users experienced a clinically significant (50%) reduction in daily hot flashes, (2) determine self-reported user outcomes regarding changes in hot flash severity, (3) describe user demographics and characteristics, (4) describe self-reported Evia app use patterns, (5) determine factors associated with intervention outcomes, and (6) determine the association of self-reported hot flash outcomes with self-reported use patterns.

## Methods

### Overview

This study is a retrospective, cross-sectional analysis of data from the Evia app collected by its developers, Mindset Health. The data were collected at 1 time point from self-report questionnaires and app use data. See Multimedia Appendix 1 for the Checklist for Reporting Results of Internet E-Surveys (CHERRIES) for this study [27]. All individuals who downloaded the app were prompted to fill out questionnaires using an open survey method (anyone could download the app and fill out the questionnaire). Evia app users were required by the app design to fill out the baseline questionnaire when they downloaded the app (before beginning the intervention program); the study authors were not provided any information regarding whether there were potential Evia app users who did not complete the baseline questionnaire and therefore did not continue the program. Participants were prompted to optionally fill out hot flash tracker logs throughout the program. All questionnaires were filled out using the Evia app. The data used as the end point measure for this study were derived from the latest hot flash log filled out by each participant. The data were collected from November 6, 2021, to February 5, 2024.

The Evia app offers a 5-week program with daily tasks such as educational readings, hypnotic inductions, and hot flash tracking. The app uses audio-recorded hypnotherapy to deliver suggestions to induce mental imagery for coolness. During the program, users can access the following hypnotherapy audio files: moving snow, cool mountain stream, rock by a lake, cooling blue light, internal thermostat, hillside breeze, snowy mountain path, walking in summer rain, eating a cool treat, cold misty lake, air-conditioned cafe, lying in a hammock, cooling face mask, frosty morning, cold day at the beach, and relaxing in a pool. Each hypnotherapy audio file has a duration of 13 to 15 minutes. App users are encouraged to listen to a hypnotherapy audio file at least once per day.

All questionnaires were developed by Mindset Health for the Evia app, and the questionnaires were in use before the time frame from which this study used data; thus, all questionnaires were provided in a way that was usable for prior app users. Over the course of this study, the user interface and intervention program remained essentially consistent. During the time span of data collection, there was a change within the Evia app in how participants were asked to report their hot flashes. One group was asked to report daily hot flashes and night sweats (hot flashes that occur at night), and a second group was asked to report only daily hot flashes (hot flashes that occur during the day). The 2 groups are discussed further, with an explanation provided for why participants were divided into 2 groups in our analysis, in the Participant Groups subsection.

### Ethical Considerations

The study protocol was reviewed by the institutional review board at Baylor University (2167670) and determined to be exempt from review because it qualified as non–human subjects research; therefore, institutional review board approval was not required. The data analyzed were provided in a deidentified form and contained no personal information. Users provided informed consent upon agreeing to the Evia app’s terms and conditions, which included notification that their deidentified data may be provided for research focused on aggregated user data rather than individual information.

### Participants

The participants are a convenience sample of women who downloaded and used the Evia app between November 6, 2021, and February 5, 2024. Given the contents of the Evia app, the respondents of the survey questionnaire are women experiencing hot flashes. A total of 835 Evia app users completed the initial questionnaire after downloading the app. Of these 835 women, 410 (49.1%) met the inclusion criteria and were included in the data analysis. Participants were included in the final analysis if they met the following criteria: (1) were aged ≥18 years and used the Evia app, defined as listening to >1 hypnotherapy audio file; (2) reported ≥3 daily hot flashes at baseline; and (3) filled out at least 1 hot flash diary log. These criteria ensured that the sample in the analysis only included women who used the Evia app past the initial download of the app, who experienced persistent hot flashes, and who had end point data to compare to baseline data. However, to better understand the differences between the people who downloaded and used the app and those who downloaded and did not use the app, demographic and characteristic data of the excluded users are also reported. Participants were not solicited or recruited for the study because the sample consists of women who downloaded and used the app. Participants provided consent digitally when downloading the Evia app.

**Figure 1:**
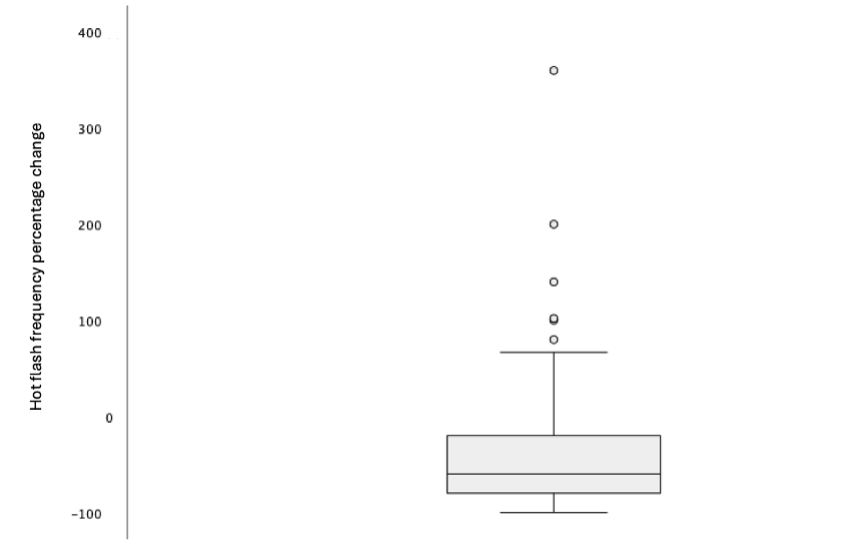
Simple box plot of hot flash frequency change outliers. After excluding app users based on the eligibility criteria, a box plot was created to view the distribution of the changes in hot flash frequency from baseline to end point. This method revealed 6 participants who were statistical outliers. To best represent the relevant data to answer our study aims, these 6 outliers were removed from the final analysis in a retrospective data analysis on app use and outcomes in women experiencing menopausal hot flashes who downloaded the Evia app between November 6, 2021, and February 5, 2024.

After excluding app users based on the eligibility criteria, a box plot (Figure 1) was used to identify outliers based on the hot flash frequency change; this method was chosen because it is a standardized way to identify outliers when the data are not normally distributed. Of the 416 participants, 6 (1.4%) were identified as outliers and removed from the analysis. We chose to remove the statistical outliers because doing so allows for better concentration on the relevant data to fulfill the study aims. This study is meant to provide an understanding of what most women experience while using the Evia app and any patterns that may be present. Removing the statistical outliers ensures that the analysis is not skewed by extreme values. This resulted in 410 (49.1%) of the 835 Evia app users who completed the initial app questionnaire being included as participants.

### Participant Groups

During the time span of data collection, the Evia app underwent several small changes to the entry questionnaire and the hot flash logs that users are prompted to respond to at the time of app download. When users downloaded the app, they were asked to report how many hot flashes they experienced. Users who downloaded the app up until June 9, 2022, were asked 2 questions about their hot flashes:

- “How many hot flashes do you experience each day?”
- “How many night sweats do you experience during the night?”

After June 9, 2022, users downloading the Evia app were only asked 1 question:

- “How many hot flashes do you experience each day?”

Due to this change in the app survey made by the developers during data collection, the 410 participants were divided into 2 groups: the first group (hot flashes and night sweats) included 139 (33.9%) women, and the second group (daily hot flashes) consisted of 271 (66.1%) women. This was necessary to accurately describe the data. This study reports the results of the 2 groups separately because their baseline hot flashes were measured differently.

### Measures

#### Aim 1

Throughout the Evia program, participants were prompted to log their daily hot flashes by entering a number in the hot flash logs. Participants in both groups were prompted to report the daily number of hot flashes, while participants with hot flashes and night sweats were prompted to report night sweats (hot flashes experienced at night) as well. In the app, the daily hot flash log was optional for users, but due to the aims of this study, only individuals who had filled out at least 1 daily hot flash log were included in the final analysis. For the participants with hot flashes and night sweats, the total number of hot flashes experienced during the day and at night were summed together for each participant at baseline and end point to provide a final number of hot flashes experienced throughout a 24-hour period. The baseline number of daily hot flashes was reported by the user in the entry questionnaire, and the end point number of daily hot flashes was the number indicated by the user on their last recorded hot flash log.

#### Aim 2

When responding to the baseline questionnaire, before beginning the app-delivered intervention, participants provided the average intensity of their hot flashes. Participants were asked, “How would you rate the intensity of your daily hot flashes (on average)?” The response options included “mild,” “moderate,” “severe,” and “very severe.” This question provided descriptions of what each level of intensity would entail. The participants were given the option of logging their daily hot flash intensity throughout the 5-week program with the hot flash tracker. The hot flash severity indicated on their last recorded hot flash log was considered the participants’ hot flash severity at end point.

#### Aim 3

In the initial questionnaire, participants filled out questions on demographics and characteristics. The order of the items on the baseline questionnaire was not randomized, and 1 item per page was displayed on the app screen, with a total of 20 pages. Throughout the questionnaire, participants were able to go back and change any answers if needed. Participants were asked to report their age and whether their menstrual cycles were “regular,” “irregular but haven’t stopped,” “stopped within the past year,” “or “stopped over a year ago.” Participants were also asked, “How would you classify your stage of menopause?” The response options included “perimenopause,” “menopause,” “postmenopause,” and “I’m not sure.” Participants were asked whether they were currently taking hormone replacement therapy and were given the options “yes,” “yes, but weaning off it,” or “no.” Participants were asked at baseline, “How do you currently manage your hot flashes?” Answer options included “natural supplements or OTC drug,” “other prescribed medications,” “hormone therapy (HRT),” “cooling tools (water spray, fans, etc),” “soy consumption (soy isoflavones),” “lifestyle changes (exercise, etc),” “antidepressants (for hot flashes),” and “other.” Participants were asked, “How important to you is a nonhormonal tool to help with hot flashes?” The answer options included “extremely important,” “somewhat important,” and “not important.” Participants were asked, “Have you tried hypnotherapy before?” The answer options were “yes” or “no.”

#### Aim 4

Each time an Evia app user listened to a hypnotherapy audio file on the app, the event was logged. To determine Evia app use patterns, this study used these listening logs to determine the number of times an individual listened to a hypnotherapy audio file as well as the number of days on which an individual listened to at least 1 audio file. Only listening events that occurred before each participant’s end point were included in the analysis.

#### Aim 5

The aforementioned characteristics and demographics, including age, menopausal stage, whether they had tried hypnosis before, the importance of a nonhormonal tool, and other intervention methods, were used as measures to determine the characteristics associated with positive outcomes in hot flash reduction.

#### Aim 6

For this aim, the listening logs for each individual will be used in conjunction with the self-reported initial daily hot flashes and the self-reported daily hot flashes indicated in the end point hot flash tracker.

### Data Analyses

In analyzing these data, descriptive statistics (means, SDs, and frequencies) were calculated for the variables of interest. For aim 1, a 2-tailed paired samples *t* test was used. Aim 2 used a Wilcoxon signed rank test. To carry out aims 5 and 6, Spearman rank correlation tests were used to test associations between the variables.

## Results

### Aim 1: Hot Flash Reduction Outcomes

This study included 410 women who used the Evia app and had ≥3 daily hot flashes. The 410 participants were divided into 2 groups: the first group (hot flashes and night sweats) included 139 (33.9%) women, and the second group (daily hot flashes) consisted of 271 (66.1%) women. At baseline, the women with hot flashes and night sweats experienced a mean of 8.330 (SD 3.977; range 3-24) daily hot flashes, while the women with daily hot flashes experienced a mean of 6.040 (SD 3.282; range 3-20) daily hot flashes. At end point, the women with hot flashes and night sweats experienced a mean of 3.070 (SD 2.883; range 0-15) daily hot flashes, while the women with daily hot flashes experienced a mean of 2.970 (SD 2.377; range 0-15) daily hot flashes. Tables 1 and 2 show the distribution, respectively, of total baseline and endpoint hot flashes reported by the group of participants with daily hot flashes and night sweats. Tables 3 and 4 show the distribution, respectively, of total baseline and endpoint hot flashes reported by the group of participants with daily hot flashes.

A paired samples *t* test indicated that there was a large, statistically significant difference in the average number of daily hot flashes experienced by the women with hot flashes and night sweats from baseline to end point (Cohen *d*=1.28; t_138_=15.055; 2-tailed *P*<.001), as well as in the average number of daily hot flashes experienced by the women with daily hot flashes from baseline to end point (Cohen *d*=.82, t_270_=13.555; 2-tailed *P*<.001).

Clinical significance in the reduction of hot flashes is defined as a reduction of 50% in daily hot flashes [5,28]. In this study, 76.3% (106/139) of the women with hot flashes and night sweats experienced at least a 50% reduction in daily hot flashes from when they completed their entry questionnaire to the latest hot flash log included in the dataset, while 56.8% (154/271) of the women with daily hot flashes experienced a clinically significant 50% reduction in daily hot flashes. The average percentage reduction in daily hot flashes for the women with hot flashes and night sweats was 61.4% (SD 33.185%), while for the women with daily hot flashes, it was 45.2% (SD 42.567%).

**Table 1:** Distribution of baseline hot flashes for women with daily hot flashes and night sweats (n=139).

Baseline total hot flashes	Participants, n (%)
3	8 (5.8)
4	10 (7.2)
5	21 (15.1)
6	15 (10.7)
7	6 (4.3)
8	13 (9.4)
9	12 (8.6)
10	39 (28.1)
11	1 (0.7)
12	1 (0.7)
13	1 (0.7)
14	0 (0)
15	5 (3.6)
16	0 (0)
17	0 (0)
18	0 (0)
19	0 (0)
20	6 (4.3)
21	0 (0)
22	0 (0)
23	0 (0)
24	1 (0.7)

**Table 2:** Distribution of end point hot flashes for women with daily hot flashes and night sweats (n=139).

End point total hot flashes	Participants, n (%)
0	22 (15.8)
1	21 (15.1)
2	28 (20.1)
3	22 (15.8)
4	17 (12.2)
5	12 (8.6)
6	3 (2.2)
7	2 (1.4)
8	4 (2.9)
9	0 (0)
10	5 (3.6)
11	0 (0)
12	1 (0.7)
13	0 (0)
14	0 (0)
15	2 (1.4)

**Table 3:** Distribution of baseline hot flashes with women with daily hot flashes (n=271).

Baseline total hot flashes	Participants, n (%)
3	30 (11.1)
4	35 (12.9)
5	135 (49.8)
6	14 (5.2)
7	3 (1.1)
8	8 (3)
9	0 (0)
10	29 (10.7)
11	0 (0)
12	2 (0.7)
13	0 (0)
14	1 (0.4)
15	8 (3)
16	1 (0.4)
17	0 (0)
18	0 (0)
19	0 (0)
20	5 (1.8)

**Table 4:** Distribution of end point hot flashes for women with daily hot flashes (n=271).

End point total hot flashes	Participants, n (%)
0	37 (13.7)
1	39 (14.4)
2	68 (25.1)
3	30 (11.1)
4	35 (12.9)
5	21 (7.7)
6	21 (7.7)
7	6 (2.2)
8	19 (7)
9	0 (0)
10	4 (1.5)
11	0 (0)
12	0 (0)
13	0 (0)
14	0 (0)
15	1 (0.4)

### Aim 2: Hot Flash Severity Outcomes

Participants were asked to report their hot flash severity at baseline and end point. For participants with hot flashes and night sweats, 2 participants declined to answer regarding their hot flash severity at baseline, 42 (30.7%) reported mild hot flashes, 65 (47.4%) moderate hot flashes, 22 (16.1%) severe hot flashes, and 8 (5.8%) very severe hot flashes. At end point, of the 139 participants with hot flashes and night sweats, 22 (15.8%) reported no hot flash severity due to the absence of hot flashes, 58 (41.7%) reported mild hot flashes, 46 (33.1%) reported moderate hot flashes, 11 (7.9%) reported severe hot flashes, and 2 (1.4%) reported very severe hot flashes.

At baseline, of the 271 participants with daily hot flashes, 73 (26.9%) reported mild hot flashes, 116 (42.8%) moderate hot flashes, 64 (23.6%) severe hot flashes, and 18 (6.6%) very severe hot flashes. At end point, of the 271 participants with daily hot flashes, 37 (13.7%) reported no hot flash severity due to the absence of hot flashes, 147 (54.2%) reported mild hot flashes, 74 (27.3%) reported moderate hot flashes, and 13 (4.8%) reported severe hot flashes. None reported very severe hot flashes.

For the participants with hot flashes and night sweats, a Wilcoxon signed rank test indicated that there was a significant (*P*<.001) difference between baseline and end point in daily hot flash severity, and the test demonstrated that 48.9% (67/137) of the women experienced a decrease in the severity of their daily hot flashes, 11.7% (16/137) had an increase in severity, and 39.4% (54/137) experienced no change in their daily hot flash severity.

For the participants with daily hot flashes, a Wilcoxon signed rank test indicated that there was a significant (*P*<.001) difference between baseline and end point in daily hot flash severity, and the test demonstrated that 64.2% (174/271) of the women experienced a decrease in the severity of their daily hot flashes, 4.4% (12/271) had an increase in severity, and 31.4% (85/271) experienced no change in their daily hot flash severity.

### Aim 3: User Demographics and Characteristics

The mean age of the participants with hot flashes and night sweats was 52.600 (SD 5.577; range 38-72) years. The mean age of the participants with daily hot flashes was 52.400 (SD 5.483; range 30-77) years. When asked to classify their stage of menopause, of the 139 participants with hot flashes and night sweats, 51 (36.7%) indicated that they were unsure, 34 (24.5%) reported that they were perimenopausal, 23 (16.5%) reported that they were menopausal, and 31 (22.3%) reported that they were postmenopausal, while of the 271 participants with daily hot flashes, 96 (35.4%) indicated that they were unsure, 86 (31.7%) reported that they were perimenopausal, 29 (10.7%) reported that they were menopausal, and 60 (22.1%) reported that they were postmenopausal. When asked about their menstrual cycles, of the 77 women with hot flashes and night sweats who responded to this question, 35 (45%) reported that their menstrual cycles had stopped more than a year ago, 18 (23%) reported that they had stopped within the past year, 20 (26%) indicated that their cycles were irregular but had not stopped, and 4 (5%) reported having regular cycles. When asked about their menstrual cycles, of the 121 women with daily hot flashes who responded to this question, 62 (51.2%) reported that their menstrual cycles had stopped more than a year ago, 30 (24.8%) reported that they had stopped within the past year, 27 (22.3%) indicated that their cycles were irregular but had not stopped, and 2 (1.7%) reported having regular cycles.

Regarding using hormone therapy for hot flashes, of the 139 participants with hot flashes and night sweats, 128 (92.1%) were not using hormone therapy, 10 (7.2%) were using hormone therapy, and 1 (0.7%) was using hormone therapy but was weaning off it, while of the 271 participants with daily hot flashes, 234 (86.3%) were not using hormone therapy, 34 (12.5%) were using hormone therapy, and 3 (1.1%) were using hormone therapy but were weaning off it. In answer to the question “How important to you is a nonhormonal tool to help with hot flashes?” of the 63 women with hot flashes and night sweats who provided an answer, 44 (70%) responded “extremely important,” 17 (27%) responded “somewhat important,” and 2 (3%) responded “not important,” while of the 150 women with hot flashes who provided an answer, 104 (69.3%) responded “extremely important,” 39 (26%) responded “somewhat important,” and 7 (4.7%) responded “not important.”

Of the 410 participants in both groups, 209 (51%) provided information at baseline on what treatments they were currently using to manage their hot flashes. Of these 209 women, 111 (53.1%) used cooling tools such as a water spray or fans, 95 (45.5%) adopted lifestyle changes such as exercise, 76 (36.4%) used natural supplements or over-the-counter drugs, 30 (14.4%) consumed soy isoflavones, 14 (6.7%) used antidepressants for hot flashes, 10 (4.8%) took other prescribed medications, and 31 (14.8%) selected “other.” The majority of the participants (136/181, 75.1%) had not tried hypnotherapy before, while the rest (45/181, 24.9%) had tried it before.

Demographics and characteristics surveyed in the baseline questionnaire were also analyzed for the 425 users who downloaded the Evia app between November 6, 2021, and February 5, 2024, but these users were not included in the full data analysis due to not using the Evia app beyond downloading it, not experiencing at least 3 daily hot flashes, or not having end point data. It is important to note that not all individuals in this group filled out all questionnaire items; therefore, the percentages are based on those who provided an answer. The mean age of these users was 51.100 (SD 6.339; range 18-83) years. When asked to classify their stage of menopause, of the 401 users who responded, 118 (29.4%) indicated that they were unsure, 151 (37.7%) reported that they were perimenopausal, 50 (12.5%) reported that they were menopausal, 79 (19.7%) reported that they were postmenopausal, and 3 (0.7%) reported that they were not menopausal. When asked about their menstrual cycles, of the 231 women who responded, 103 (44.6%) reported that their menstrual cycles had stopped more than a year ago, 42 (17.7%) reported that they had stopped within the past year, 61 (26.4%) indicated that their cycles were irregular but had not stopped, and 25 (10.8%) reported having regular cycles. Regarding using hormone therapy for hot flashes, of the 419 users who responded, 362 (86.4%) were not using hormone therapy, 45 (10.7%) were using hormone therapy, and 12 (2.9%) were using hormone therapy but were weaning off it. In answer to the question “How important to you is a nonhormonal tool to help with hot flashes?” of the 166 users who provided an answer, 94 (56.6%) responded “extremely important,” 58 (34.9%) responded “somewhat important,” and 14 (8.4%) responded “not important.” Not all participants responded to this question because it was added to the Evia questionnaire partway through the data collection period. The majority of the participants (96/146, 65.8%) had not tried hypnotherapy before, while the rest (50/146, 34.2%) had tried it before.

### Aim 4: Evia App Use Patterns

Evia app use can be evaluated by the number of days on which a user listened to at least 1 hypnotherapy audio file. Participants’ end point data, based on their last logged hot flash diary, varied in the time elapsed since baseline (when the app was downloaded). For the participants with hot flashes and night sweats, end point ranged from day 1 (baseline) to day 730, with the average length of time from baseline being 110.31 (SD 152.86) days. For the participants with daily hot flashes, end point ranged from day 1 (baseline) to day 461, with the average length of time from baseline being 67.62 (SD 78.25) days. This differed from the number of days on which the participants used the app. The average number of distinct days on which the participants with hot flashes and night sweats listened to at least 1 audio file was 35.370 (SD 44.638; range 1-307). The average number of hypnotherapy audio files listened to overall by the participants with hot flashes and night sweats was 40.380 (SD 50.741; range 2-344). The average number of distinct days on which the participants with daily hot flashes listened to at least 1 audio file was 27.140 (SD 35.663; range 1-390). The average number of hypnotherapy audio files listened to overall by the participants with daily hot flashes was 31.280 (SD 39.816; range 2-414).

### Aim 5: Factors Associated With Outcomes

The factors tested for association with the Evia app users’ outcomes were participants’ age, menopausal stage, hormone use, and past hypnotherapy use. A Spearman rank correlation test on the data of the participants with hot flashes and night sweats found no significant associations between participants’ age, menopausal stage, hormone use, or past hypnotherapy use and the outcomes in hot flash frequency reduction. The results of the correlation test are shown in Table 5.

**Table 5:** Spearman rank correlation test for user characteristics and hot flash frequency percentage reduction among the participants with hot flashes and night sweats^a^.

Characteristics	Correlation coefficient
Age (n=139)	–0.029
Menopausal stage (n=139)	0.027
Hormone use (n=139)	–0.113
Past hypnotherapy use (n=31)	0.070

^a^This table shows the correlational analysis results for the associations between user characteristics and user outcomes in a retrospective data analysis on app use and outcomes in women who experience menopausal hot flashes and downloaded the Evia app between November 6, 2021, and June 9, 2022.

A Spearman rank correlation test on the data of the participants with daily hot flashes found no significant associations between participants’ age, hormone use, or past hypnotherapy use and their outcomes in hot flash frequency reduction. However, there was a small, positive, significant correlation between participants’ menopausal stage and percentage reduction in hot flashes. The results of the correlation test are shown in Table 6.

**Table 6:** Spearman rank correlation test for user characteristics and hot flash frequency percentage reduction among the participants with daily hot flashes (n=271)^a^.

Characteristics	Correlation coefficient
Age	–0.092
Menopausal stage	0.239^b^
Hormone use	0.047
Past hypnotherapy use	0.051

^a^This table shows the correlational analysis results for the associations between user characteristics and user outcomes in a retrospective data analysis on app use and outcomes in women who experience menopausal hot flashes and downloaded the Evia app between June 10, 2022, and February 5, 2024.

^b^*P*<.001.

### Aim 6: Use Pattern Associations With Outcomes

A Spearman rank correlation test to assess the association between app use and percentage reduction in hot flashes among the participants with hot flashes and night sweats found a small, positive, statistically significant association between the number of days on which a user listened to at least 1 hypnotherapy audio file and the percentage reduction in hot flashes from baseline to end point. There was also a small, positive, statistically significant association between the total number of hypnotherapy audio files that users listened to and the percentage reduction in hot flashes from baseline to end point. The results of the correlation test are presented in Table 7.

**Table 7:** Spearman rank correlation test for app use and hot flash frequency percentage reduction among the participants with hot flashes and night sweats (n=139)^a^.

App use	Correlation coefficient
Number of distinct days listened	0.209^b^
Number of audio file listens	0.199^c^

^a^This table shows the correlational analysis results for the associations between user outcomes and users’ app use in a retrospective data analysis on app use and outcomes in the women with hot flashes and night sweats who experience menopausal hot flashes and downloaded the Evia app between November 6, 2021, and June 9, 2022. There was a small significant correlation between increased app use and a greater percentage reduction in hot flashes.

^b^*P*=.01.

^c^*P*=.02.

A Spearman rank correlation test to assess the association between app use and percentage reduction in hot flashes among the participants with daily hot flashes found no significant association between the number of days on which they listened to at least 1 hypnotherapy audio file and the percentage reduction in hot flashes from baseline to end point. There was also no statistically significant association between the total number of hypnotherapy audio files that users listened to and the percentage reduction in hot flashes from baseline to end point. The results of the correlation test are presented in Table 8.

**Table 8:** Spearman rank correlation test for app use and hot flash frequency percentage reduction among the participants with daily hot flashes (n=271)^a^.

App use	Correlation coefficient
Number of distinct days listened	0.091
Number of audio file listens	0.080

^a^This table shows the correlational analysis results for the associations between user outcomes and users’ app use in a retrospective data analysis on app use and outcomes in the women with daily hot flashes who experience menopausal hot flashes and downloaded the Evia app between June 10, 2022, and February 5, 2024. There were no significant correlations between increased app use and a percentage reduction in hot flashes.

## Discussion

### Principal Findings

The results demonstrated that the majority of Evia app users experienced a clinically significant reduction of at least 50% in their daily hot flashes, with 76.3% (106/139) of the participants with hot flashes and night sweats and 56.8% (154/271) of the participants with daily hot flashes achieving mean reductions of 61.4% and 45.2%, respectively. The difference in daily hot flashes from baseline to end point was statistically significant (*P*<.001) for both groups. This result demonstrates the potential benefits of the Evia app. Although this result should be interpreted in light of the fact that there was no control group in this dataset to compare with, the majority of women who began using the Evia app saw a decrease in their hot flashes of at least 50%. Using the measure of clinical significance to interpret these results will allow clinicians to provide clear information when discussing how the Evia app might benefit their patients. The participants with hot flashes and night sweats had a greater proportion reaching clinical significance due to more accurate reporting of hot flashes experienced during a 24-hour period (at day and night); thus, this group had a higher reported baseline mean of hot flashes than the participants with daily hot flashes. Although the baseline mean number of daily hot flashes for both groups differed (participants with hot flashes and night sweats: mean 8.330, SD 3.977; participants with daily hot flashes: mean 6.040, SD 3.282) because the participants with daily hot flashes were not being asked to report night sweats separately, both groups had a similar mean number of daily hot flashes at end point (participants with hot flashes and night sweats: mean 3.070, SD 2.883; participants with daily hot flashes: mean 2.970, SD 2.377).

In this study, many Evia app users also experienced decreases in their hot flash severity. Hot flash severity decreased in 48.9% (67/137) of the women with hot flashes and night sweats and 64.2% (174/271) of the women with daily hot flashes. Crucially, participants’ severe and very severe hot flashes decreased: of the 410 women across both groups, 86 (21%) reported severe hot flashes at baseline, but only 24 (5.9%) reported severe hot flashes at end point; similarly, at baseline, 26 (6.3%) reported very severe hot flashes, but only 2 (0.5%) reported very severe hot flashes at end point. This result demonstrates that even women who do not experience a clinically significant reduction in their hot flash frequency may still experience significant benefit by a decrease in hot flash severity. The significant decreases in both hot flash frequency and severity are encouraging due to the need for accessible and nonpharmacological interventions for menopausal hot flashes. These results warrant further investigation in a randomized controlled trial of the Evia app to better understand if this is an efficacious mobile delivery method for hypnotherapy in treating menopausal hot flashes.

This study found a small, positive, significant association between app use and hot flash outcomes among the participants with hot flashes and night sweats. This may indicate that the more an individual uses the Evia app hypnotherapy audio files, the greater the percentage reduction in hot flashes. However, the correlation was small; therefore, this result should be interpreted with caution. In addition, there was not a similarly significant result among the participants with daily hot flashes. App use varied widely between participants (ranging from 1 to 390 unique days), and this study did not evaluate whether participants used the app on consecutive days. As the Evia program is designed to be used daily for 5 weeks, it may be that some participants’ lack of consistency made it challenging to understand the effects of use on outcome. A future study may be able to address this by examining within a more standardized study design whether use is associated with hot flash outcomes. Furthermore, this dataset relied on single-day measures of hot flash frequency for both baseline and end point. This may also have impacted the lack of a significant association between use and outcomes because women will naturally have some variability in their hot flash frequency from day to day.

Likewise, the lack of significant associations between participant characteristics such as age, hormone use, and past hypnotherapy use and outcomes is likely due to the lack of standardization across participants. There was a small, positive, significant association between participants’ menopausal stage and hot flash reduction among the participants with daily hot flashes, with women further along in menopausal stage being more likely to have greater hot flash reductions. However, this association should be interpreted with caution, given that it is a small correlation within a large sample size, and there was no control group; no significant correlation was found among the participants with hot flashes and night sweats. A future study should further investigate what characteristics might be associated with achieving a clinically significant outcome through a randomized controlled trial design.

When comparing the users who met the inclusion criteria with users who did not continue using the app, the demographics and characteristics were generally comparable between the groups. One difference is that the mean number of daily hot flashes (6.820, SD 3.691) among the included users (n=410) at baseline was higher than the mean number of daily hot flashes (3.310, SD 3.799) among the excluded users (n=425). It may be that those who experienced more daily hot flashes were more likely to persist in using the app beyond the initial download.

### Comparisons With Prior Work

In the retrospective study by Snyder and Elkins [26] on the demographics and characteristics of Evia app users, the authors indicated that a surprising number of women (3048/9103, 33.48%) reported that they were unsure of their menopausal stage. Our study found this proportion to be slightly higher when looking at the sample included in the analysis, with 35.9% (147/410) indicating that they were unsure of their menopausal stage. There may be a knowledge gap in the general population of women on the topic of menopause. An increase in educational content on menopausal stages in the Evia app may provide a needed resource for women.

This study found the mean number of daily hot flashes to be 8.330 (SD 3.977) among the participants with hot flashes and night sweats and 6.040 (SD 3.282) among the participants with daily hot flashes at baseline, both of which are lower than those found in an earlier clinical trial conducted by Elkins et al [21], where the authors found the mean number of daily hot flashes to be 10. However, the distribution of the baseline hot flash frequency is consistent with that in previous studies, with more women experiencing ≥5 hot flashes [21,26]. This may be due to the differences in measuring hot flashes between this study and the one by Elkins et al [21]. Our study had a single-item measure, with participants being asked, “How many hot flashes do you experience each day?” In the study by Elkins et al [21], participants tracked their daily hot flashes for a week, which was then averaged, thus providing a more accurate method due to women experiencing variable numbers of hot flashes from day to day. Future studies of the Evia app should explore the use of a weekly hot flash log.

As our study did not have a control group for comparison, we lack the ability to know how a control group’s hot flash frequency and severity might have changed over time compared to the intervention group. However, by comparing our findings to those of prior research that included control groups, we can better understand the typical pattern of hot flash severity and frequency over time. Prior research has demonstrated that there is not a large change in hot flash frequency or severity over time for a control group. A 2013 randomized controlled trial examining face-to-face hypnosis for hot flashes found that the control group’s hot flash score (a product of hot flash frequency × hot flash severity) only demonstrated a mean reduction of 8.32% compared to the mean reduction of 71.36% in the hot flash score of the hypnosis group [21]. Future studies should examine Evia app users compared to a matched control to understand whether the outcomes are significantly different.

### Limitations

A primary limitation of this study concerns the change in the baseline questionnaire that resulted in 1 group of Evia app users not being specifically asked about their night sweats. This meant that the participants with daily hot flashes might have underreported their total number of hot flashes experienced during a 24-hour period (at day and night). However, we still observed that both the participants with hot flashes and night sweats and those with daily hot flashes had a very similar mean number of daily hot flashes at end point (3.070, SD 2.883 and 2.970, SD 2.377, respectively). In addition, since the completion of the data collection for this study, the Evia app has been updated to once again ask users specifically about night sweats, separate from daytime hot flashes.

Another primary limitation of this study is the lack of end point data for information collected in the baseline survey. Due to the retrospective nature of this analysis, participants only completed a baseline survey, after which they filled out hot flash tracker logs throughout the program; as a result, only hot flash frequency and severity could be compared between baseline and end point.

The data collected included the number of times a user listened to a hypnotherapy audio file. Listens were only logged in the app once a user had completed listening to the audio file. Thus, there are no data captured for users who may have partially listened to a hypnotherapy audio file. While the intervention is designed to have users listen to the hypnotherapy audio files in their entirety, future studies might consider including partial listens to the data collected.

In addition, this study is unable to fully assess the impacts of potential participant attrition. Participants who were more motivated to use all elements of the app, such as the hot flash diary logs (which served as end point measures), may have experienced greater benefit. Alternatively, those who benefited from the intervention may have reduced app use due to the absence of ongoing hot flashes. Given this limitation, future replication of this study is warranted.

Finally, this study was also limited by the lack of standardization across participants. Due to the retrospective nature of this analysis, the data included participants with varying levels of Evia app use. Some of the participants completed the 5-week Evia program, while others did not; some of the participants used the Evia app frequently, while others did not. A future study with a greater level of standardization across participants would be able to better provide a clearer understanding of the benefits of the Evia app.

### Future Directions

Considering the positive clinical outcomes noted in this study, there is a need for future studies to address this study’s limitations by conducting a randomized controlled trial of the Evia app for menopausal hot flashes. A randomized controlled trial will be able to clearly demonstrate whether the Evia app is an effective mobile delivery method for hypnotherapy in treating menopausal hot flashes. The randomized controlled trial should compare the Evia intervention to a placebo control. A future study should administer postintervention measures to provide end point data, ensure that all participants complete the 5-week program to reduce the variability in use among participants, and use weekly hot flash diaries to gain a more accurate measure of hot flash frequency. In addition, a randomized controlled trial of the Evia app should examine when during the 5-week program women begin to see results because this study only examined baseline and end point data.

### Conclusions

This study is the first to report the hot flash frequency and severity outcomes of Evia app users. The results demonstrated that 76.3% (106/139) of the Evia app users who were asked about hot flashes during the day and night experienced a clinically significant reduction in their daily hot flashes, while 56.8% (154/271) of the Evia app users who were only asked about daily hot flashes achieved a clinically significant reduction in their daily hot flashes. In addition, 48.9% (67/137) of the women with hot flashes and night sweats and 64.2% (174/271) of the women with daily hot flashes experienced a decrease in their hot flash severity from baseline to end point. This study demonstrates the potential benefits of the Evia app as an accessible and nonpharmacological intervention for menopausal hot flashes. However, additional research is needed to explore the efficacy of the app in randomized controlled trial as well as to better understand the factors that moderate and mediate beneficial therapeutic outcomes.

## Appendix

Multimedia Appendix 1 Checklist for Reporting Results of Internet E-Surveys (CHERRIES).

## Abbreviations

CHERRIES: Checklist for Reporting Results of Internet E-Surveys

## Data Availability

The datasets generated and analyzed during this study are available from the corresponding author on reasonable request.
