# Supplementary material for: User Outcomes for an App-Delivered Hypnosis Intervention for Menopausal Hot Flashes: Retrospective Analysis
Source: JMIR Form Res. 2025 Jan 9;9:e63948. doi: 10.2196/63948 (PMC11757980; doi:10.2196/63948)
Supplement: Multimedia Appendix 1 [file formative_v9i1e63948_app1.docx]

**Table 1.** Checklist for Reporting Results of Internet E-Surveys (CHERRIES)

| Item category | Checklist item | Explanation | Manuscript reference |
| --- | --- | --- | --- |
| **Design** |  |  |  |
|  | Describe survey design | Describe target population, sample frame. Is the sample a convenience sample? | p. 6–7 |
| **Institutional Review Board (IRB) approval and informed consent process** |  |  |  |
|  | IRB approval | Mention whether the study has been approved by an IRB | p. 6 |
|  | Informed consent | Describe the informed consent process. | p. 6 |
|  | Data protection | If any personal information was collected or stored, describe what mechanisms were used to protect unauthorized access. | p. 6 |
| **Development and pre-testing** |  |  |  |
|  | Development and testing | State how the survey was developed, including whether the usability and technical functionality of the electronic questionnaire had been tested before fielding the questionnaire. | p. 5–6 |
| **Recruitment process and description of the sample having access to the questionnaire** |  |  |  |
|  | Open survey versus closed survey | An “open survey” is a survey open for each visitor of a site, while a closed survey is only open to a sample which the investigator knows. | p. 5 |
|  | Contact mode | Indicate whether the initial contact with the potential participants was made on the Internet. | p. 7 |
|  | Advertising the survey | How/where was the survey announced or advertised? | p. 7 |
| **Survey administration** |  |  |  |
|  | Web/email | State the type of e-survey. If it is an email survey, were the responses entered manually into a database, or was there an automatic method for capturing responses? | p. 5 |
|  | Context | Describe the website in which the survey was posted. What is the website about, who is visiting it, what are visitors normally looking for? | p. 4–5 |
|  | Mandatory/voluntary | Was it a mandatory survey to be filled in by every visitor who wanted to enter the website, or was it a voluntary survey? | p. 5 |
|  | Incentives | Were any incentives offered? | N/A |
|  | Time/date | In what timeframe were the data collected? | p. 5 |
|  | Randomization of items or questionnaires | To prevent biases items can be randomized or alternated. | p. 9 |
|  | Adaptive questioning | Use adaptive questioning to reduce number and complexity of the questions. | N/A |
|  | Number of items | What was the number of questionnaire items per page? | p. 9 |
|  | Number of screens (pages) | Over how many pages was the questionnaire distributed? | p. 9 |
|  | Completeness check | It is technically possible to do consistency or completeness checks before the questionnaire is submitted. Was this done, and if “yes,” how (usually JAVAScript)? An alternative is to check for completeness after the questionnaire has been submitted (and highlight mandatory items). | The information needed to determine completeness prior to submission of the questionnaires was not provided to us as a part of the dataset from the app development company. However, all users completed the baseline questionnaires in full. |
|  | Review step | State whether respondents were able to review and change their answers. | p. 9–10 |
| **Response rates** |  |  |  |
|  | Unique site visitor | If you provide view rates or participation rates, you need to define how you determined a unique visitor. | Each person who downloaded the app was assigned a unique user ID based on cookies. The cookies allowed all subsequent activity from each unique user to be collected. |
|  | View rate (ratio of unique survey visitors/unique site visitors) | Requires counting unique visitors to the first page of the survey, divided by the number of unique site visitors. | The information needed to determine this was not provided to us as a part of the dataset from the app development company. |
|  | Participation rate (ratio of unique visitors who agreed to participate/unique first survey page visitors) | Count the unique number of people who filled in the first survey page, divided by visitors who visit the first page of the survey. | The information needed to determine this was not provided to us as a part of the dataset from the app development company. |
|  | Completion rate (ratio of users who finished the survey/users who agreed to participate) | The number of people submitting the last questionnaire page, divided by the number of people who agreed to participate. | The information needed to determine this was not provided to us as a part of the dataset from the app development company. |
| **Preventing multiple entries from the same individual** |  |  |  |
|  | Cookies used | Indicate whether cookies were used to assign a unique user identifier to each client computer. If so, mention the page on which the cookie was set and read, and how long the cookie was valid. | Cookies were used to assign a unique user identifier to each app user. The cookie was set when users agreed to the terms and conditions when they first downloaded the app. The cookies were active during the whole course of users’ app usage. |
|  | IP check | Indicate whether the IP address of the client computer was used to identify potential duplicate entries from the same user. | Evia does not use IP addresses to identify unique users. |
|  | Log file analysis | Indicate whether other techniques to analyze the log file for identification of multiple entries were used. If so, please describe. | No other methods used. |
|  | Registration | In “closed” (non-open) surveys, users need to login first and it is easier to prevent duplicate entries from the same user. Describe how this was done. | N/A because this study used data from an open survey. |
| **Analysis** |  |  |  |
|  | Handling of incomplete questionnaires | Were only completed questionnaires analyzed? | p. 5 |
|  | Questionnaires submitted with an atypical timestamp | Some investigators may measure the time people needed to fill in a questionnaire and exclude questionnaires that were submitted too soon. Specify the timeframe that was used as a cut-off point and describe how this point was determined. | N/A |
|  | Statistical correction | Indicate whether any methods such as weighting of items or propensity scores have been used to adjust for the non-representative sample. | N/A |

*Note*. Based on Eysenbach’s (2004) CHERRIES method [28]. This table describes the author’s adherence to a checklist for ensuring the quality of reporting the results for an Internet e-survey study as applied to a retrospective data analysis on app usage and outcomes in women who experience menopausal hot flashes and downloaded the Evia app from October 2021 to February 2024.
